# Puerarin: A Potential Therapeutic for Colon Adenocarcinoma (COAD) Patients Suffering From SARS-CoV-2 Infection

**DOI:** 10.3389/fphar.2022.921517

**Published:** 2022-05-23

**Authors:** Weizheng Liang, Xiushen Li, Yue Yao, Qingxue Meng, Xueliang Wu, Hao Wang, Jun Xue

**Affiliations:** ^1^ Central Laboratory, The First Affiliated Hospital of Hebei North University, Zhangjiakou, China; ^2^ Department of General Surgery, The First Affiliated Hospital of Hebei North University, Zhangjiakou, China; ^3^ Department of Obstetrics and Gynecology, Shenzhen University General Hospital, Shenzhen, China; ^4^ Guangdong Key Laboratory for Biomedical Measurements and Ultrasound Imaging, School of Biomedical Engineering, Shenzhen University Health Science Center, Shenzhen, China; ^5^ Shenzhen Key Laboratory, Shenzhen University General Hospital, Shenzhen, China; ^6^ Department of Internal Medicine of Traditional Chinese Medicine, The First Clinical Medical School, Guangzhou University of Chinese Medicine, Guangzhou, China

**Keywords:** puerarin, COVID-19, COAD, comorbidity, network pharmacology, bioinformatics

## Abstract

Patients with colonic adenocarcinoma (COAD) are at relatively high risk of SARS-CoV-2 infection. However, there is a lack of medical strategies to treat COVID-19/COAD comorbidity. Puerarin, a natural product, is a known antiviral, antitumor, and immunomodulatory effect. Therefore, we hypothesised that puerarin could be used to treat COVID-19/COAD patients. Based on network pharmacology and bioinformatics analysis, the potential targets and pharmacological mechanisms of puerarin in COVID-19/COAD were identified. By intersecting therapeutic target genes for puerarin, COVID-19-related genes and COAD-related genes, 42 target genes of puerarin that could potentially treat COVID-19/COAD comorbidity were obtained. By using the 42 potential target genes to construct the protein-protein interaction (PPI) network, we obtained five core target genes, namely RELA, BCL2, JUN, FOS, and MAPK1. The results of bioinformatics analysis revealed that puerarin could be able to treat COVID-19/COAD comorbidity through apoptosis, antiviral, antioxidant, NF-κB signaling pathway, MAPK signaling pathway, IL-17 signaling pathway, TNF signaling pathway, and HIF-1 signaling pathway etc. This study found that puerarin has the potential to treat COVID-19/COAD patients and that the therapeutic target genes obtained in the study may provide clues for the treatment of COVID19/COAD comorbidity.

## Introduction

Since the emergence of the SARS-CoV-2 virus, COVID-19 has spread rapidly worldwide. The latest data from the World Health Organization show that the cumulative number of confirmed cases and deaths worldwide has been more than 500 million and six million (updated on 05 May 2022). With a global daily average of about 500,000 patients with COVID-19, the situation remains critical for epidemic prevention. Similar to SARS-CoV, SARS-CoV-2 was a highly pathogenic coronavirus, which were mainly transmitted through aerosols or droplets, leading to the development of respiratory diseases and even death (Xu et al., 2020). Due to the small size of droplets and aerosols carrying the SARS-CoV-2 virus, it is difficult to achieve the expected preventive effect (Jayaweera et al., 2020). The common clinical symptoms in most SARS-CoV-2 infected patients were cough and fever, with partial patients also presenting with sore throat, diarrhea, and loss of taste (Giacomelli et al., 2020; Li et al., 2020). However, a few of patients with COVID-19 probably developed acute respiratory failure or acute respiratory distress syndrome, which might even lead to death (Chen et al., 2020). The emergence of the SARS-CoV-2 virus has caused enormous economic and health losses to people around the world. Through the continuous efforts of scientists from around the world, several vaccines and drugs have been used for COVID-19 (Liu et al., 2020; Lazarus et al., 2021). However, there was no significant change in the number of additional COVID-19 patients per day.

Both malignant tumor and COVID-19 are major public health problems worldwide. Colorectal cancer, a common gastrointestinal malignancy, had the third-highest incidence and mortality rate of all cancers in the United States, for both men and women (Siegel et al., 2022). With the rapid development of modern medicine, significant breakthroughs have been made in basic research, early diagnosis, chemotherapy, and targeted therapy for colorectal cancer, yet the survival rate of colorectal cancer patients has not been significantly improved (Durinikova et al., 2021). Colon adenocarcinoma (COAD) is a common malignant tumor among all types of colon cancer, with a high mortality rate and high recurrence rate (Liu, 2021). Due to the lack of specific early diagnostic symptoms and markers, most patients were already at an advanced stage at the time of diagnosis, and the high metastatic rate characteristic of advanced COAD was a major factor in the high mortality rate of COAD patients (Brown and Solomon, 2018). Therefore, exploring the mechanism of COAD pathogenesis, progression, and metastasis and finding effective biomarkers and therapeutic targets can greatly help to improve the survival rate of COAD patients. The latest study found that the vast majority of patients with malignancies are chronically immunosuppressed and therefore more susceptible to SARS-CoV-2 infection than healthy individuals, often resulting in a poorer prognosis (Kamboj and Sepkowitz, 2009; Liang et al., 2020). If patients with COAD are infected with the SARS-CoV-2 virus, it may lead to a worse prognosis. Therefore, it is necessary to find drugs that can be used to treat COVID-19/COAD patients.

Traditional Chinese medicine (TCM), derived from more than 2,000 years of clinical experience, has been a crucial role in the treatment of a variety of epidemics and has made significant contributions to the health of the Chinese people. With the outbreak of COVID-19, combinations of TCM treatment protocols have been widely used in China, achieving better clinical outcomes. In China, more than four-fifths of COVID-19 patients have received TCM treatment (Ren et al., 2020). Pueraria Lobata, one of the commonly used TCMs, has be reportedly used for anti-inflammatory, immunomodulatory, anti-cancer and so on (Wang et al., 2020). Puerarin, as the main active ingredient of Pueraria Lobata, has similar pharmacological effects to Pueraria Lobata. As a potent inhibitor of influenza virus neuraminidase, puerarin could reduce influenza virus titers, decrease respiratory inflammatory responses and diminish mortality (Wang et al., 2021). Puerarin nanosuspension showed good anticancer activity and low toxicity and has the potential to become a therapeutic medicine for patients with COAD (Wang et al., 2013). On this basis, we hypothesized that puerarin might be used for COVID-19/COAD patients. Therefore, network pharmacology and other bioinformatic methods were employed to explore the therapeutic targets and pharmacological mechanisms of puerarin in COVID-19/COAD and to elucidate the potential therapeutic value of puerarin.

## Materials and Methods

### Flowchart of the Study

**FIGURE 1:**
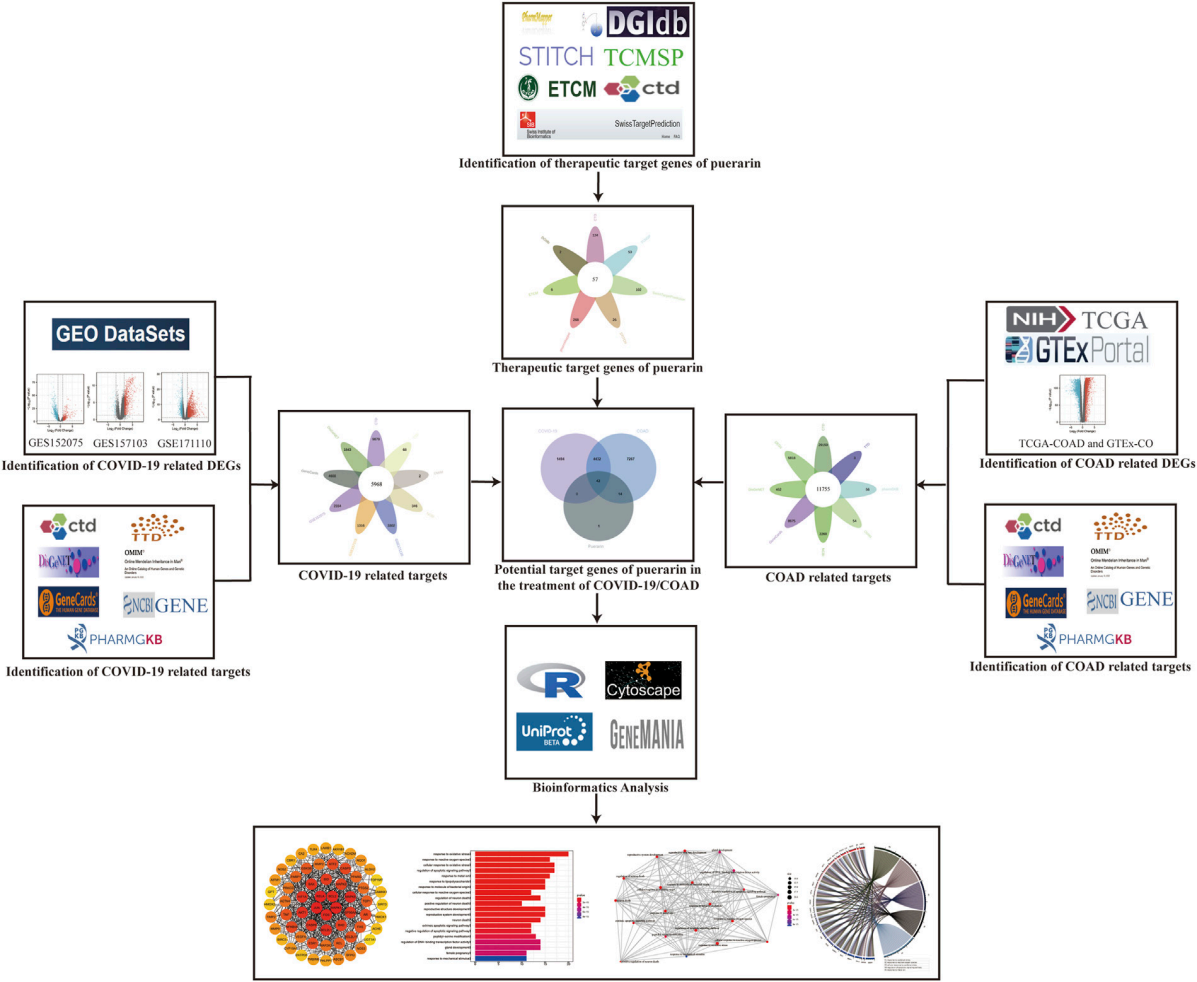
Flowchart. This study followed the analytical flow of the flowchart to explore the target genes and molecular mechanisms of puerarin in the treatment of COAD/COVID-19.

Figure 1 depicts the entire flow of this study. Firstly, targets associated with COVID-19 were identified though the Gene Expression Omnibus (GEO) dataset and multiple disease-target related databases; then targets associated with COAD were identified by using the TCGA-COAD dataset, the GTEx-colon data dataset, and multiple disease-target related databases; next, multiple drug-target related databases were used to find the therapeutic targets of puerarin. The COVID-19-related targets, COAD-related targets, and therapeutic targets of Puerarin were intersected. Finally, bioinformatics analysis was used to explore the pharmacological mechanisms of puerarin in COVID-19/COAD comorbidity.

### Identification of Targets Associated With COVID-19 or COAD

Transcriptome sequencing data from the COVDID-19-related datasets (GSE157103, GSE152075, GSE171110) were downloaded though the GEO database (https://www.ncbi.nlm.nih.gov/geo/). The differentially expressed genes (DEGs) associated with COVDID-19 were filtered according to the filtering criteria (*p* < 0.05 and absolute value of log fold change >1) by using the “limma” package in R. In addition, we also searched seven authoritative databases containing CTD (http://ctdbase.org/), OMIM (https://omim.org/), DisGeNET (https://www.disgenet.org/), GeneCards (https://www.genecards.org), NCBI (https://www.ncbi.nlm.nih.gov/), TTD (http://db.idrblab.net/), pharmaGKB (https://www.pharmgkb.org/) to further explore genes associated with COVID-19. Gene names obtained from the different databases were harmonized. We considered the genes that appeared at least twice as COVID-19-related genes.

Transcriptome sequencing data from the TCGA-COAD and GTEx-CO datasets were obtained via the UCSC Xena (http://xena.ucsc.edu/) database. DEGs associated with COAD (*p* < 0.05 and absolute value of log fold change >1) were screened by the R language “limma” package. In addition, CTD, OMIM, DisGeNET, GeneCards, NCBI, TTD, and pharmaGKB databases were used to obtain COAD-associated genes. We considered the genes that appeared at least twice as COAD-related genes.

### Identification of Puerarin Therapeutic Targets

To fully explore the therapeutic targets of puerarin, we searched seven authoritative databases related to drug targets: 1) PharmMapper (http://www.lilab-ecust.cn/pharmmapper/); 2) CTD (http://ctdbase.org/); 3) SwissTargetPrediction (http://www.swisstargetprediction.ch/); 4) Drug Gene Interaction Database (DGIdb, https://www.dgidb.org/); 5) TCMSP (https://tcmspw.com/tcmsp.php); 6) STITCH (http://stitch.embl.de/); 7) ECTM (http://www.tcmip.cn/ETCM/). To ensure the accuracy of subsequent analyses, targets that appeared at least twice in the database were considered to be therapeutic target genes for puerarin.

### Construction of PPI Network and Analysis of Core Genes

We referred to the intersection of therapeutic target genes for puerarin, COVID-19-related genes and COAD-related genes as target genes of puerarin in COVID-19/COAD comorbidity. The protein-protein interaction (PPI) network was constructed by using the GeneMANIA database (http://genemania.org/). The GeneMANIA database is one of the recognized bioinformatics databases that helps to explain the molecular mechanisms involved in the target proteins. Cytoscape software was used to visualize the degree of target genes and to identify the core genes for puerarin treatment of COVID-19/COAD.

### Bioinformatics Analysis

The uniport database was used to convert gene names to gene IDs for subsequent bioinformatic analysis. We called the “enrichGO” and “enrichKEGG” functions in R to perform Gene Ontology (GO) and Kyoto Encyclopedia of Genes and Genomes (KEGG) pathway enrichment analysis on the target genes for puerarin treatment of COVID-19/COAD, and the “ggplot2” package in R to visualise the analysis results in R (only displayed the 20 results with the lowest q values). Finally, we used the “circlize” package in R to construct the circos plots showing the results of the top five GO and KEGG enrichment analyses with the corresponding genes.

## Results

### Identification Results of COVID-19-Related and COAD-Related Targets

**FIGURE 2:**
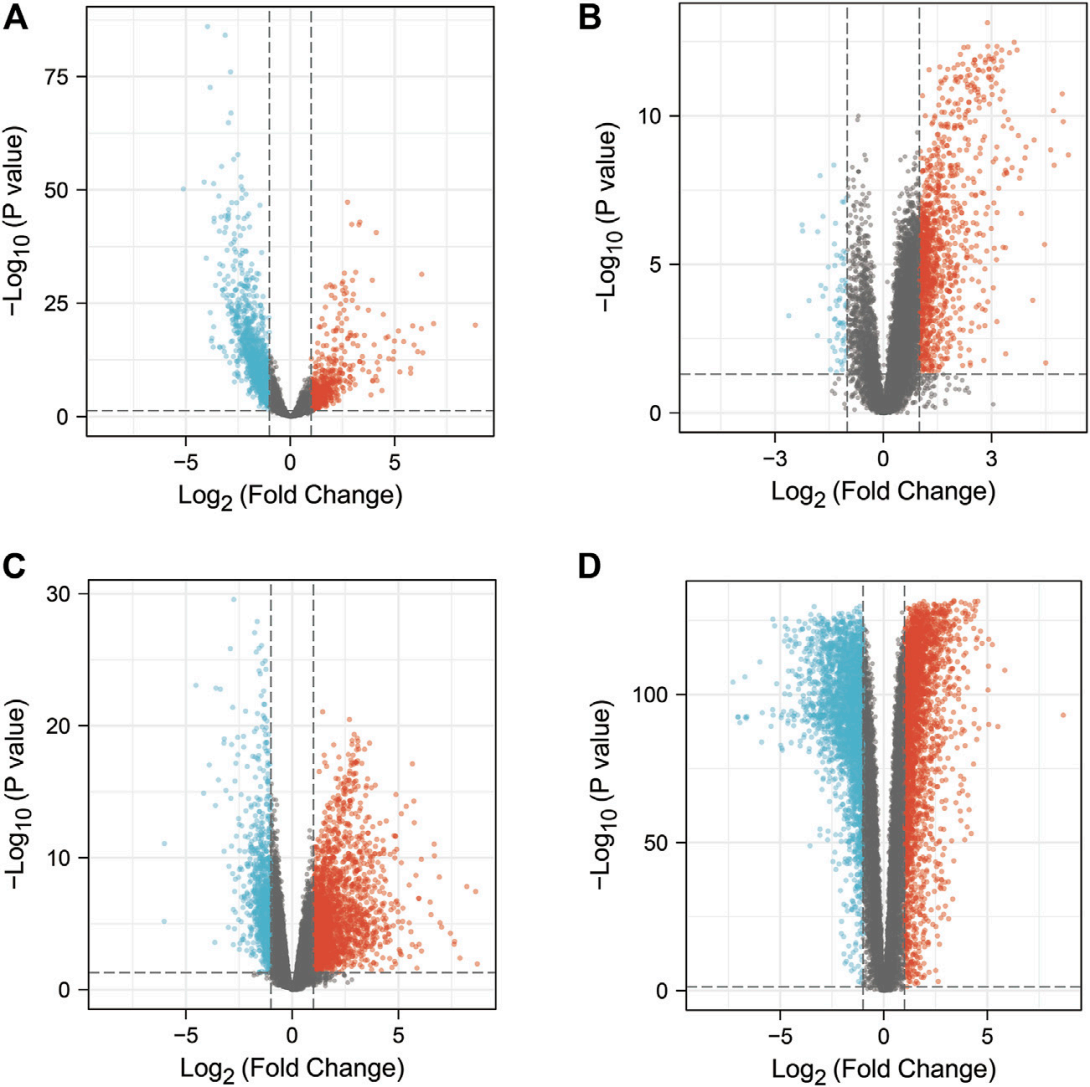
DEGs from COAD patients or COVID-19 patients (*p* < 0.05 and absolute value of log fold change >1). The red and blue dots represented genes with increased or decreased expression in COVID-19 patients or COAD patients, respectively, while the black dots represented genes with no significant difference in expression between COVID-19 or COAD patients. **(A)** Volcano plot of DEGs in the GSE152075 dataset. **(B)** Volcano plot of DEGs in the GSE157103 dataset. **(C)** Volcano plot of DEGs in the GSE171110 dataset. **(D)** Volcano plot of DEGs in the TCGA-COAD dataset and the GTEx-CO dataset.

**FIGURE 3:**
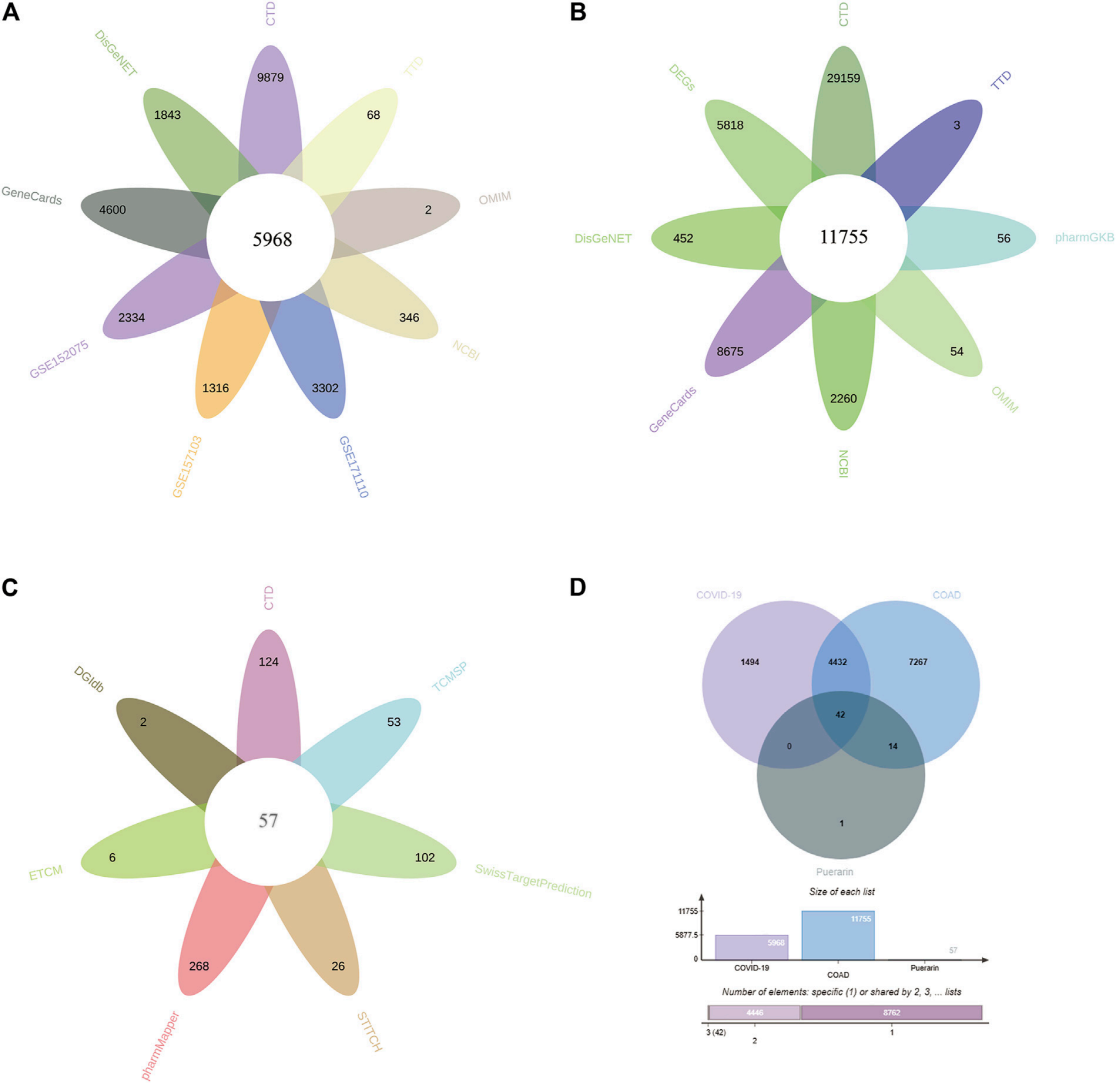
Potential target genes of puerarin in the treatment of COVID-19/COAD comorbidity. **(A)** Genes associated with COVID-19 from 3 GEO datasets and six databases. **(B)** Genes associated with COAD from the TCGA-COAD dataset, GTEx-CO dataset, and seven databases. **(C)** Therapeutic target genes of Puerarin from seven databases. **(D)** Venn diagram of therapeutic target genes for puerarin, COVID-19-related genes and COAD-related genes.

The study contained 3 GEO datasets associated with COVID-19: GSE152075, GSE157103, and GSE171110. In the GSE157103 dataset, we included sequencing data from 484 nasopharyngeal swab samples (containing 430 COVID-19 positive patients and 54 COVID-19 negative healthy individuals). In the GSE157103 dataset, we included sequencing data from 126 plasma samples (containing 100 COVID-19 positive patients and 26 COVID-19 negative healthy individuals). In the GSE171110 dataset, we included sequencing data from 54 whole blood samples (containing 44 COVID-19 positive patients and 10 COVID-19 negative healthy individuals). The DEGs in the GEO dataset were searched though the “limma” package in R. 2334, 1316, 3302 DEGs were obtained from the GSE152075, GSE157103, GSE171110 datasets respectively (Figures Figure2A–C). In addition, CTD, OMIM, DisGeNET, GeneCards, NCBI, TTD, and pharmaGKB databases were used to find 9,879, 2, 1843, 4,600, 346, 68, and 0 genes associated with COVID-19, respectively (Figure Figure3A, Supplementary Table S1). In the current study, we considered target genes that appeared in at least two databases to be COVID-19-related genes, resulting in 5968 COVID-19-related genes.

In this study, the TCGA and GTEx datasets were used to identify DEGs in COAD patients. We combined the data from TCGA-COAD and GTEx-colon and included sequencing data from a total of 349 normal colon samples and 471 COAD samples. The data were analyzed by using the “limma” package in R, resulting in 5,818 DEGs (Figure Figure2D). Through CTD, OMIM, DisGeNET, GeneCards, NCBI, TTD, and pharmaGKB databases, we obtained 29,159, 54, 452, 8,675, 2,260, 3, 56 genes associated with COAD (Figure Figure3B, Supplementary Table S2). We referred to target genes that appeared in at least two databases as COAD-related genes, resulting in 11,755 COAD-related genes.

### Identification Results of Puerarin Target Genes

To obtain therapeutic target genes for puerarin, seven databases containing PharmMapper, CTD, SwissTargetPrediction, DGIdb, TCMSP, STITCH, and ECTM were searched, resulting in 268, 124, 102, 2, 53, 26, and six therapeutic target genes respectively (Figure 3C, Supplementary Table S3). The therapeutic target genes were screened by using the presence of at least two databases as a screening condition, resulting in 57 therapeutic targets for puerarin.

### Therapeutic Target Genes of Puerarin in COVID-19/COAD

We intersected the therapeutic target genes of puerarin, COAD-related genes, and COVID-19-related genes, and presented them by using Venn diagram (Figure 3D, Supplementary Table S4). We defined these 42 intersecting genes as gene set 1 and used them for subsequent bioinformatics analysis.

### PPI Network

**FIGURE 4:**
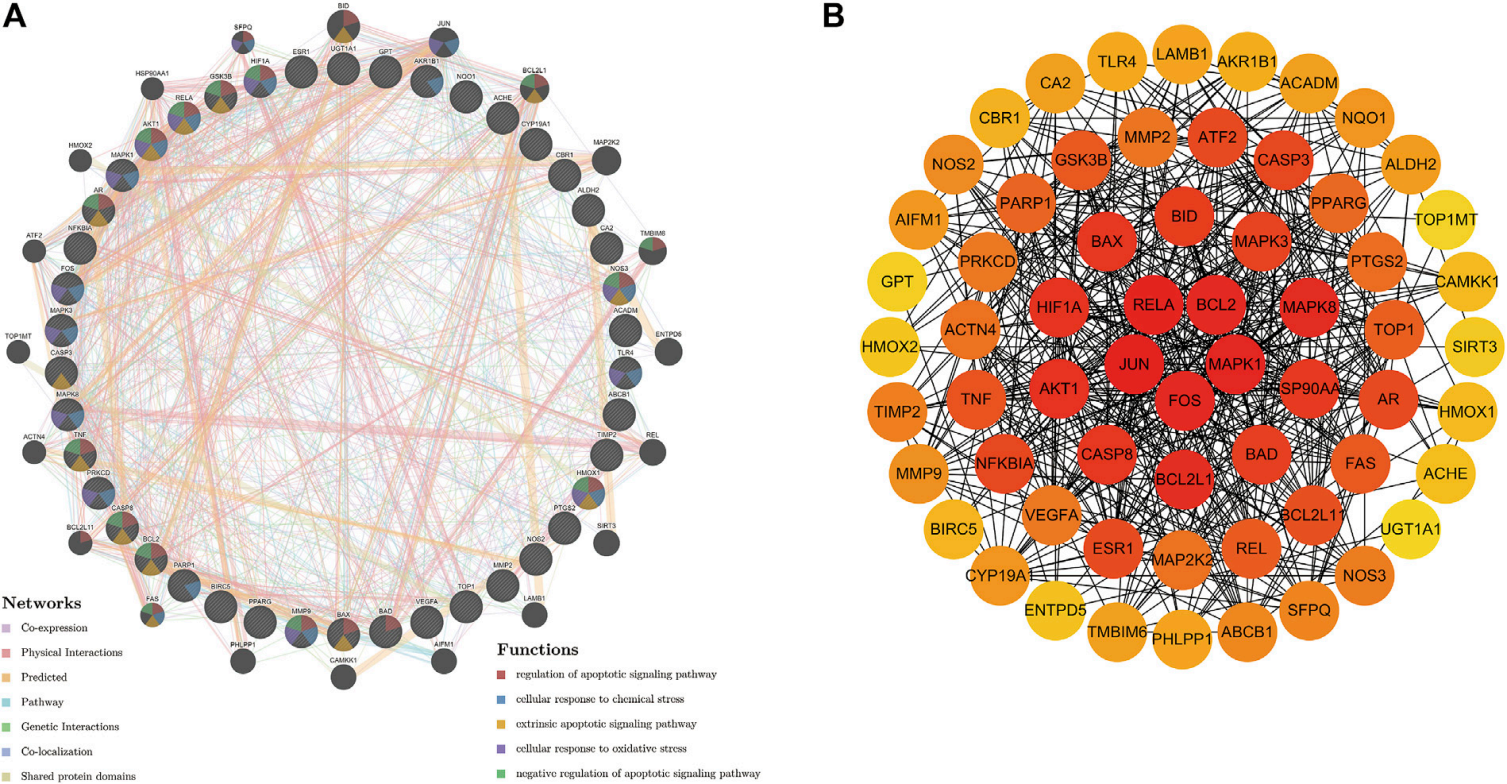
PPI network of potential therapeutic target genes of puerarin in the treatment of COVID-19/COAD. **(A)** The PPI network was derived from the GeneMANIA database. The different relationships between nodes were indicated by the different colored connecting lines. Nodes were enriched in different functions indicated by the colors of the nodes. **(B)** PPI networks constructed from nodal degree values. The degree of the node was proportional to the depth of the node color.

We used 42 genes from gene set 1 for PPI network construction. The GeneMANIA database, based on large amounts of genomics and proteomics data, is commonly used for the prediction of gene function and the construction of interactions between genes. In this study, we uploaded gene set 1 to the GeneMANIA database. The results of the analysis were shown in Figure 4A (Supplementary Table S5). The circular nodes represented genes, and the different colors of the node represented gene enriched in different functional analysis results. The different colored lines between the nodes represented the different interactions. The mechanism of puerarin in COVID-19/COAD comorbidity might be related to apoptotic, chemical stress, and oxidative stress. By using Cytoscape software to visualize the degree of each gene in the PPI network (Figure 4B), we found that RELA, BCL2, JUN, FOS, and MAPK1 had the highest degree, suggesting that the five genes might be core targets of puerarin in COVID-19/COAD comorbidity.

### Results of Bioinformatics Analysis

**FIGURE 5:**
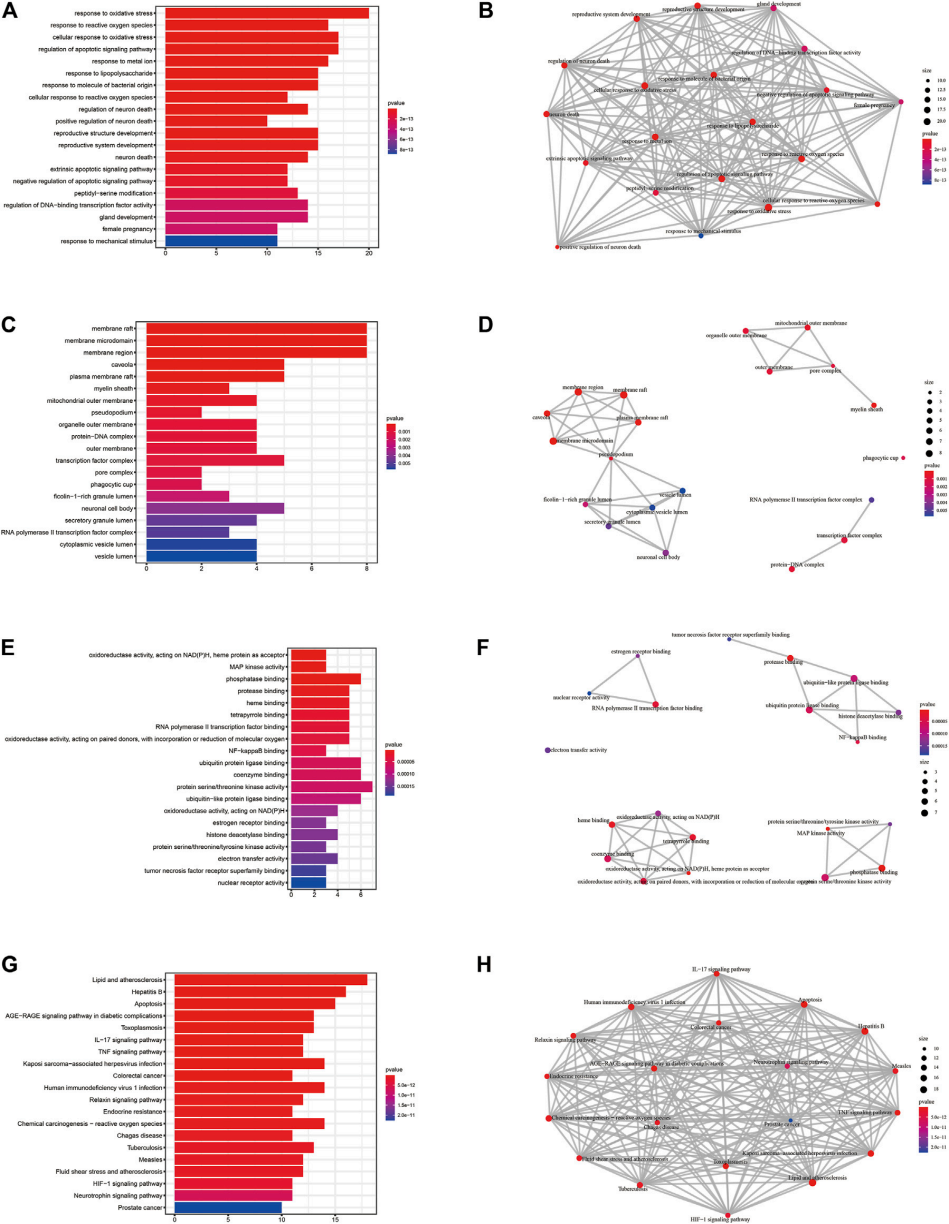
GO and KEGG enrichment analysis of potential therapeutic target genes of puerarin for COVID-19/COAD (only the 20 pathways with the smallest *p*-values were shown). **(A)**, **(C)** and **(E)** The results of BP, CC, and MF term enrichment analysis, respectively. **(B)**, **(D)** and **(F)** The results of correlation analysis of BP, CC, MF term enrichment analysis, respectively. **(G)** The results of KEGG enrichment analysis. **(H)**The results of correlation analysis of KEGG enrichment analysis.

**FIGURE 6:**
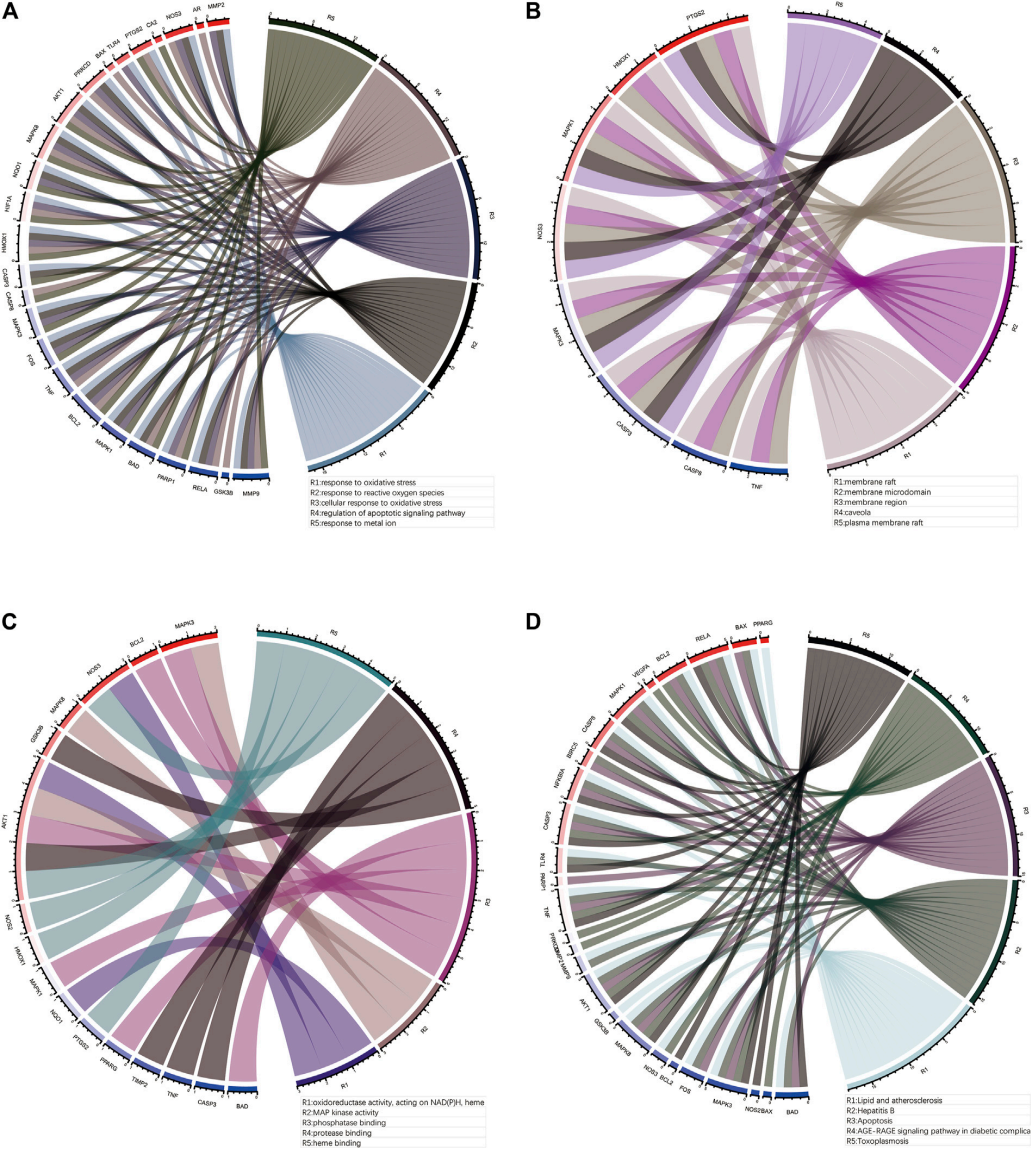
Top five enrichment analysis results and corresponding genes. **(A)**, **(B)** and **(C)**The top five results of BP, CC, MF enrichment analysis and corresponding genes. **(D)** The top five results of KEGG enrichment analysis and the corresponding genes.

To investigate the pharmacological mechanism of puerarin in the treatment of COVID-19/COAD, we performed bioinformatic analysis of 42 genes in gene set 1. The “cluster profile” package in R was used to perform enrichment analysis of the BP, CC, and MF aspects of GO (Figures 5A–C, Supplementary Table S6). The results of the top five BP, CC, MF, and KEGG enrichment analyses and the corresponding genes were presented using circus plots (Figures 6A–D). The results of the BP enrichment analysis mainly included regulation of apoptotic signaling pathway, response to the metal ion, response to lipopolysaccharide, response to molecule of bacterial origin, regulation of neuron death, productive system development, peptidyl-serine modification, and so on. The results of the CC enrichment analysis found that gene set 1 was mainly involved in caveola, plasma membrane raft, myelin sheath, mitochondrial outer membrane, pseudopodium, organelle outer membrane, protein-DNA complex, and so on. The results of the MF enrichment analysis found that gene set 1 mainly involved MAP kinase activity, phosphatase binding, NF-κB binding, ubiquitin-protein ligase binding, coenzyme binding, histone deacetylase binding, tyrosine kinase activity, etc.

In addition, the results of the KEGG pathway enrichment analysis mainly included Apoptosis, Colorectal cancer, IL-17 signaling pathway, TNF signaling pathway, Human immunodeficiency virus 1 infection, HIF-1 signaling pathway (Figure 5D, Supplementary Table S7).

## Discussion

The SARS-CoV-2 virus has been spreading globally for more than 2 years and has not yet shown signs of improvement, yet we still lack effective drugs against COVID-19. However, in China, protocols that combine Chinese medicine with Western medicine to treat patients with COVID-19 have achieved excellent results. Clinical studies have found that TCM treatment can reduce the rate of severe disease, decrease the use of antibiotics and alleviate clinical symptoms including fever and cough (Huang et al., 2021; Zhang et al., 2021). Cumulatively, one in twenty people worldwide has been infected with the SARS-CoV-2 virus, and patients with COAD are more susceptible to SARS-CoV-2 virus infection than healthy individuals due to their chronically depressed immune system. Based on the antiviral and anticancer properties of puerarin, we combined bioinformatics and network pharmacology approaches to investigate the target genes and mechanisms of puerarin in the treatment of COVID-19/COAD (Wei et al., 2014).

We analyzed data from open databases and defined the 42 potential target genes of puerarin in the treatment of COVID-19/COAD as gene set 1. Through the GeneMANIA database to construct a PPI network and perform functional analysis, we found that gene set 1 mainly has the functions of chemical stress, apoptosis, and oxidative stress. Chemical stress in the body might trigger adverse drug reactions, and adverse reactions during the treatment of COAD patients often led to the inability of patients to tolerate subsequent treatment (Mutter et al., 2015). Apoptosis isthe normal physiological process that occurs within the body in a state of relative equilibrium. Disruption of this balance may lead to disease. A high number of apoptotic cells, for example, could lead to degenerative disorders, whereas a low number of apoptotic cells could contribute to tumor growth and progression (Wong, 2011). But apoptosis is a double-edged sword, and induction of apoptosis in tumor cells is one of the therapeutic mechanisms for tumor patients. Abnormal differentiation and apoptosis of T cells, one of the hallmarks of severe COVID-19, led to immune dysfunction in patients (Leonardi and Proenca, 2020). Persistent oxidative stress might lead to chronic inflammation, which in turn led to the development of diseases including cancer, lung disease, and diabetes (Reuter et al., 2010). Studies have confirmed that oxidative stress is positively related to the incidence of COAD (Barrett et al., 2017). SARS-CoV-2 virus induces the abnormal oxidative stress response in humans, which in turn can lead to the development of the respiratory syndrome and even death in patients. Therefore, drugs that improve the oxidative stress status of patients may improve prognosis and reduce mortality. We screened the genes based on their degree values and identified RELA, BCL2, JUN, FOS, and MAPK1 as possible core target genes for puerarin in treatment of COVID-19/COAD. The NF-κB signaling pathway is involved in several biological processes such as cellular transformation, and immune response in the body. Abnormal NF-κB directly contributed to tumorigenesis, growth, and metastasis, while NF-κB activity was controlled by phosphorylation of upstream genes containing RELA (Lu and Yarbrough, 2015). Inhibition of NF-κB pathway activity reduced the likelihood of severe disease in COVID-19 patients (Hariharan et al., 2021). As an important factor regulating apoptosis, BCL2 has been associated with the development and progression of cancer and autoimmune diseases, and several drugs targeting BCL2 have been developed (Siddiqui et al., 2015). Clinical studies have found that serum concentrations of BCL2 in COVID-19 patients correlate with patient prognosis, with high BCL2 concentrations predicting a better prognosis (Lorente et al., 2021). Activator protein 1, consisting of Jun-Jun dimer or Jun-Fos dimer, regulated the transcription of multiple genes required for biological processes and the development and progression of many tumors, including COAD (Ashida et al., 2005). As one of the complexes of activator protein 1, c-Jun was involved in the crosstalk, integration, and amplification of intercellular signaling pathways, which were involved in cellular activities including survival, apoptosis, proliferation, and tumorigenesis (Meng and Xia, 2011). MAPK signaling pathway controlled cell survival, senescence, and drug resistance by integrating extracellular signals (Braicu et al., 2019). MAPK1 was found to play an important role in the invasion and proliferation of COAD cell line SW480 (Yang et al., 2018). The severe inflammatory response that occurred in severe COVID-19 patients might be associated with activation of the p38 MAKP signaling pathway (Grimes and Grimes, 2020).

The results of bioinformatics analysis revealed that the mechanism of puerarin in COVID-19/COAD might be associated with NF-κB, MAPK, IL-17, TNF, and HIF-1 signaling pathway. Elevated pro-inflammatory cytokines, including IL-17 and TNF-α, were the main causes of disease deterioration in COVID-19 patients (Shibabaw, 2020). IL-17 was involved in the activation of multiple signaling pathways resulting to the emergence of multiple cytokines (e.g. TNF-α, IL-1β) and chemokines, which in turn led to cytokine storm (Ryzhakov et al., 2011; Veldhoen, 2017; Badawi, 2020). Inflammatory cytokines IL-17 and TNF-α upregulated PD-L1 expression in COAD cell line HCT116 through activation of NF-κB signaling pathways, leading to COAD progression (Wang et al., 2017). HIF-1 induced an adaptive response in tumor cells under localized hypoxic conditions in cancer patients and was associated with poor patient prognosis (Lu et al., 2016). Activation of HIF signaling pathway was related to tumor cell invasion and tumor cell drug resistance (Brocato et al., 2014). HIF-1α signaling pathway was involved in SARS-CoV-2 virus infection and subsequent inflammatory response, often leading to poor prognosis in COVID-19 patients (Tian et al., 2021).

## Conclusion

In this study, a series of bioinformatics analysis were employed to explore that the potential treatment mechanisms of puerarin in COVID-19/COAD comorbidity. These mechanisms may be related to apoptosis, antiviral, antioxidant, IL-17, TNF, and HIF-1 signaling pathway. The present study provides the preliminary basis and directions for the study of puerarin in COVID-19/COAD. However, as the study is based on transcriptome sequencing data and multiple databases, experimental validation is still lacking. In the follow-up studies, we will use new coronavirus-infected immune cell lines to co-culture with COAD cell lines. The therapeutic effect of puerarin will be observed by simulating the *in vivo* condition of COVID-19/COAD patients.

## Data Availability

The datasets presented in this study can be found in online repositories. The names of the repository/repositories and accession number(s) can be found in the article/Supplementary Material.

## Supplementary Material

Supplementary Table S1 Therapeutic target genes of Puerarin.

Supplementary Table S2 COVID-19-related genes.

Supplementary Table S3 COAD-related genes.

Supplementary Table S4 Potential target genes of puerarin in the treatment of COVID-19/COAD.

Supplementary Table S5 The results of PPI network.

Supplementary Table S6 The results of GO enrichment analysis.

Supplementary Table S7 The results of KEGG enrichment analysis.

## Abbreviations

COAD, Colon Adenocarcinoma; PPI, Protein-Protein Interaction; TCM, Traditional Chinese Medicine; GEO, Gene Expression Omnibus; DEGs, Differentially Expressed Genes; GO, Gene Ontology; KEGG, Kyoto Encyclopedia of Genes and Genomes.
